# *PTPRF *is disrupted in a patient with syndromic amastia

**DOI:** 10.1186/1471-2350-12-46

**Published:** 2011-03-31

**Authors:** Surasawadee Ausavarat, Siraprapa Tongkobpetch, Verayuth Praphanphoj, Charan Mahatumarat, Nond Rojvachiranonda, Thiti Snabboon, Thomas C Markello, William A Gahl, Kanya Suphapeetiporn, Vorasuk Shotelersuk

**Affiliations:** 1 Interdepartment of Biomedical Sciences, Faculty of Graduate School, Chulalongkorn University, Bangkok, 10330, Thailand; 2 Center of Excellence for Medical Genetics, Department of Pediatrics, Faculty of Medicine, Chulalongkorn University, Bangkok, 10330, Thailand; 3 Molecular Genetics Diagnostic Center, King Chulalongkorn Memorial Hospital, Thai Red Cross, Bangkok, 10330, Thailand; 4 Center for Medical Genetics Research, Rajanukul Institute, Bangkok, 10400, Thailand; 5 Department of Surgery, Faculty of Medicine, Chulalongkorn University, Bangkok, 10330, Thailand; 6 Department of Internal Medicine, Faculty of Medicine, Chulalongkorn University, Bangkok, 10330, Thailand; 7 Section on Human Biochemical Genetics, Medical Genetics Branch, National Human Genome Research Institute, National Institutes of Health, Bethesda, Maryland, 20892, USA

**Keywords:** amastia, athelia, development of breasts and nipples, ectodermal dysplasia, renal agenesis, balanced chromosome translocation, *PTPRF*, *LAR*

## Abstract

**Background:**

The presence of mammary glands distinguishes mammals from other organisms. Despite significant advances in defining the signaling pathways responsible for mammary gland development in mice, our understanding of human mammary gland development remains rudimentary. Here, we identified a woman with bilateral amastia, ectodermal dysplasia and unilateral renal agenesis. She was found to have a chromosomal balanced translocation, 46,XX,t(1;20)(p34.1;q13.13). In addition to characterization of her clinical and cytogenetic features, we successfully identified the interrupted gene and studied its consequences.

**Methods:**

Characterization of the breakpoints was performed by molecular cytogenetic techniques. The interrupted gene was further analyzed using quantitative real-time PCR and western blotting. Mutation analysis and high-density SNP array were carried out in order to find a pathogenic mutation. Allele segregations were obtained by haplotype analysis.

**Results:**

We enabled to identify its breakpoint on chromosome 1 interrupting the *protein tyrosine receptor type F gene *(*PTPRF*). While the patient's mother and sisters also harbored the translocated chromosome, their non-translocated chromosomes 1 were different from that of the patient. Although a definite pathogenic mutation on the paternal allele could not be identified, *PTPRF*'s RNA and protein of the patient were significantly less than those of her unaffected family members.

**Conclusions:**

Although *ptprf *has been shown to involve in murine mammary gland development, no evidence has incorporated *PTPRF *in human organ development. We, for the first time, demonstrated the possible association of *PTPRF *with syndromic amastia, making it a prime candidate to investigate for its spatial and temporal roles in human breast development.

## Background

Excellent progress has been made in defining the signaling pathways responsible for mammary gland development in mice [1], current knowledge about human mammary gland development is however very restricted and requires further elucidation. This may be related to the extreme rarity of absence of breast or amastia, with only about 62 patients reported in the literature (Additional file 1, Table S1). Amastia has likely been selected against in human evolution, but its occurrence provides an invaluable means to identify genes involved in human breast development.

Amastia is the complete absence of breast which is the result of complete failure of mammary ridge to develop at about 6 weeks in utero [2]. In addition, there is a lack of breast development during puberty whereas other secondary sexual characters and fertility are normal [2]. Amastia can be isolated or syndromic. Syndromes associated with the absence of breasts and nipples include ectodermal dysplasia of the tricho-odonto-onychial type (MIM# 129510), acral-renal-ectodermal-dysplasia-lipoatrophic-diabetes (AREDYLD syndrome) (MIM# 207780), and the scalp-ear-nipple syndrome (SEN or Finlay-Marks syndrome) (MIM# 181270). The latter is the most common amastia-associated syndrome. Additionally, renal involvement has been reported in some cases of scalp-ear-nipple syndrome [3,4]. In familial cases of amastia, both autosomal dominant and autosomal recessive inheritances have been reported (Additional file 1, Table S1).

Here we reported an 18-year-old female with syndromic amastia who had a reciprocal balanced translocation, 46, XX, t(1;20)(p34.1;q13.13). In addition to characterization of her clinical and cytogenetic features, we successfully identified the interrupted gene and studied its consequences.

## Methods

### Clinical descriptions

**Figure 1:**
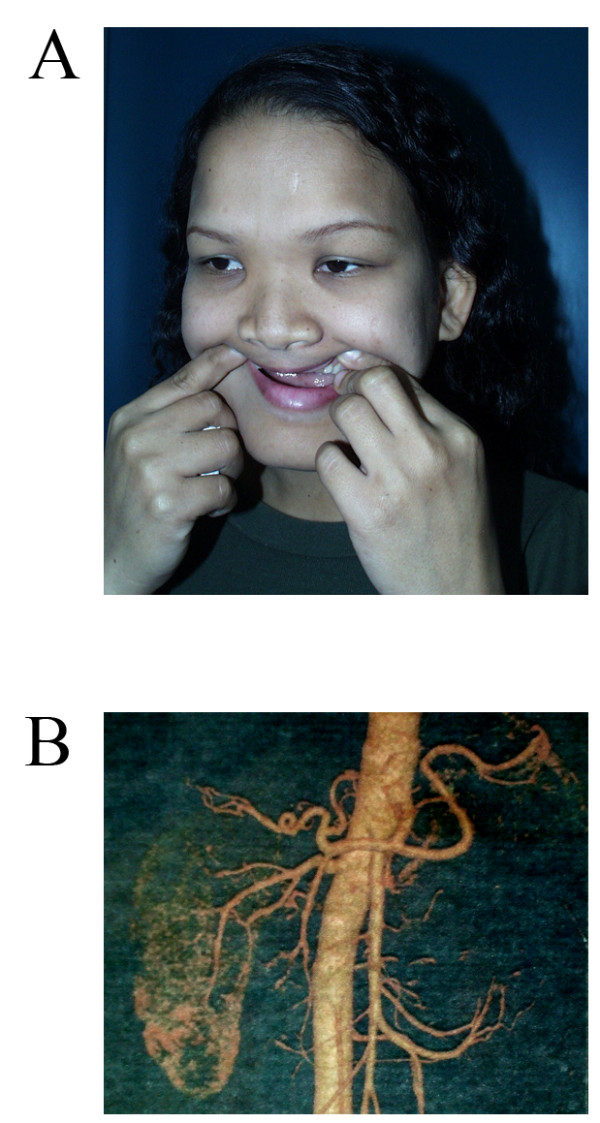
**Clinical features of the proband**. (A) Face shows absence of all four upper incisors (small, brown and easily decayed) after extraction at age 15 years, epicanthal folds, and small cup-shaped pinnae. (B) Computerized tomography of kidneys shows absence of the left kidney and its renal artery.

We identified an 18-year-old Thai woman who presented to plastic surgeons for total breast reconstruction. Menarche occurred at age 14 years and menstruation was regular. She had been generally healthy with normal intelligence. Height was normal (157 cm, 50th centile). Blood pressure was 140/90 mmHg. The patient had epicanthal folds, small and cup-shaped pinnae, absence of all four upper incisors (small, brown and easily decayed) after extraction at age 15 years (Figure 1A), bilateral absence of breasts and nipples, normal pectoralis muscles, brittle nails and normal external genitalia. Ultrasonography and computed tomography of the kidneys revealed absence of the left kidney and left renal artery, yet normal right kidney and uterus (Figure 1B). A renal function study by a post captopril Tc-99 mMAG3 test showed normal right kidney function with no demonstrable left kidney. The patient was the third child with an elder brother, an elder sister and a younger sister. Her father was deceased. No other family member was affected.

### Karyotype Analysis

Peripheral blood samples from the patient and her family members were obtained after written informed consent. Metaphase chromosomes were obtained from phytohemagglutinin (PHA)-stimulated peripheral blood lymphocytes. G-banding was performed using standard methods. The karyotype was at a resolution of 550 bands.

### Fluorescence in situ hybridization (FISH)

Cell suspensions from the phytohemagglutinin (PHA)-stimulated peripheral blood lymphocytes were used in all FISH experiments. Probes mapping to the region of the cytogenetically determined breakpoints were selected from the Mapviewer NCBI http://www.ncbi.nlm.nih.gov/mapview/ and obtained from the BACPAC Resources Center (BPRC, Oakland, CA) (Additional file 1, Table S2). BACs, PACs, or long-range PCR (10 kb each) products were labeled by nick translation with Spectrum Green or Spectrum Orange according to manufacturer's protocols (Abbott Molecular/Vysis, Des Plains, IL). Labeled probes were denatured and hybridized to metaphase spreads on the microscope slide.

### Generation of FISH probes using long-range PCR

Three primer pairs were chosen from the genomic sequence of breakpoint-spanning clone on chromosome 1 (RP5-1029K14) (Additional file 1, Table S3). Each probe was overlapped and used for subsequent FISH analysis. We used 200 ng of plasmid DNA, 1X Amplibuffer C (Vivantis, Oceanside, CA), 160 mM (NH_4_)_2_SO_4_, 500 mM Tris-HCl,17.5 mM MgCl_2 _and stabilizers, 0.4 mM dNTPs, 0.4 μM of each primer and 2.5 U Perpetual OptiTaq DNA polymerase (Vivantis, Oceanside, CA) in a total volume of 50 μl. The PCR amplification was performed as follows: initial denaturation at 95°C for 15 minutes, followed by 40 cycles of denaturation at 95°C for 30 seconds. The annealing step was performed at optimal annealing temperature for each specific primer for 30 seconds. The extension was at 72°C for 11 minutes and the final extension was at 72°C for 10 minutes.

### Analysis of repetitive elements within the breakpoint region

We screened DNA sequences for interspersed repeats and low complexity DNA sequences in the breakpoint region using RepeatMasker available at http://www.repeatmasker.org/.

### Establishment of Ebstein-Barr virus immortalization of human B-lymphocytes

We isolated Peripheral Blood Mononuclear Cells (PBMCs) using Ficoll-Parque gradient. Peripheral blood lymphocytes (B-lymphocytes) were immortalized by infecting them with active Epstein Barr Virus supernatant [5]. Cyclosporin A was added to suppress the growth of the T cells present in the preparation. Half of the supernatant was replaced by fresh medium once a week. Transformation occurred within 2 to 3 weeks after starting the culture. The human immortalized cells were seeded with the primary culture medium (RPMI1640 with 20% fetal bovine serum, 100 units/ml of penicillin-streptomycin) and were cultured in 37°C with 5% CO_2_.

### Quantitative Real-Time PCR

*PTPRF *RNA level was quantified by StepOnePlus™ Real-Time PCR Systems (Applied Biosystems, Foster City, CA). Total RNA extracted from EBV transformed lymphoblastoid cell lines was converted to cDNA using the SuperScript III First-Strand Synthesis System for RT-PCR (Invitrogen, Carlsbad, CA). *PTPRF *RNA levels were studied by quantitative real-time analysis (qRT), using two different TaqMan probes. The first one, Hs00160858_m1 (Applied Biosystems, Foster city, CA), spanning exons 5 and 6, is 5' to the chromosomal breakpoint, while the other, Hs00892984_m1 (Applied Biosystems), spanning exons 32 and 33, is 3' to the breakpoint. The probes were run in triplicate in separate tubes. Relative expression analysis was calculated in terms of ΔΔCt normalized to *GAPDH *transcript levels, using forward primer; 5'-GTGAAGGTCGGAGTCAACGG-3', reverse primer; 5'-TCAATGAAGGGGTCATTGATGG-3' and probe; HEX-CGCCTGGTCACCAGGGCTGC-BHQ1.

### Western Blot Analysis

Lymphoblastoid protein was extracted using ice-cold RIPA lysis buffer with Halt protease inhibitor cocktail (Pierce, Roxford, IL). Protein concentration was determined using the BCA protein assay reagent (Pierce, Roxford, IL). Western blot was performed in triplicate. Protein extract of 100 μg was electrophoresed, transferred to PVDF membrane and incubated with 1:100 anti-PTPRF (BD Transduction Laboratory, San Jose, CA) and 1:1000 goat anti-mouse IgG-HRP: sc-2005 (Santa Cruz Biotechnology, Inc., Santa Cruz, CA). The membrane was stripped and reprobed with 1:1000 anti-GAPDH (Trevigen, Gaitherburgs, MD) and 1:1000 goat anti-rabbit IgG-HRP: sc-2030 (Santa Cruz Biotechnology, Inc., Santa Cruz, CA). PTPRF expression was normalized with GAPDH and compared with that of a control.

### Mutation analysis

Total RNA and genomic DNA were extracted from peripheral blood using Qiagen RNA and DNA extraction kits according to manufacturer's instructions, respectively (Qiagen, Valencia, CA). Reverse transcription was performed using ImProm-II™ reverse transcriptase (Promega, Madison, WI), according to the manufacturer's instructions. PCR amplification of the entire coding sequence of the *PTPRF *gene, its promoter [6] (-654 to + 294) and cDNA amplification was performed using set of primers and parameters (Additional file 1, Table S4). In brief, we used 50 ng of either genomic DNA or 100 ng of cDNA, 1×PCR buffer (Promega, Madison, WI), 1.5 mM MgCl2, 0.2 mM dNTPs, 0.2 μM of each primer and 0.5 U Taq DNA polymerase (Promega) in a volume of 20 μl. Amplification of the promotor sequences of the *PTPRF *gene was performed in a reaction mixture of 1× Qiagen PCR buffer (containing 1.5 mM MgCl_2_), 1× Q-Solution, 0.1 unit HotStarTaq (Qiagen), 200 μM dNTPs, 0.2 μM of each primer and 50 ng of genomic DNA. PCR products were treated with ExoSAP-IT (USP Corporation, Cleveland, OH) according to the manufacturer's recommendations, and sent for direct sequencing (Macrogen Inc., Seoul, Korea). Sequence data were analyzed using Sequencher (version 4.2; Gene Codes Corporation, Ann Arbor, MI).

### High-density SNP genotyping array

We performed SNP array for genome wide genotyping using Illumina Human 1 M BeadArray. SNP genotyping was performed according to manufacturer's protocols. The results include within-sample normalized fluorescence ('x' and 'y'), between-sample normalized fluorescence ('Log R ratio' and 'B-allele frequency') and SNP genotype calling. We analyzed data using Bead Studio software and PennCNV. The PennCNV package is available at http://www.openbioinformatics.org/penncnv.

### Haplotype analysis

Panels 1 and 2 of the ABI Prism Linkage Mapping Set version 2.5 (Applied Biosystems, Foster City, CA) were amplified. These two panels consist of 31 tandem repeat markers on chromosome 1 that define a 10 cM resolution human index map. Each panel consists of a group of markers that were fluorescently labeled with FAM, HEX, or NED. Amplifications were carried out as single reactions in 96 well plates, according to the manufacturer's protocols. An additional four microsatellite markers selected from Mapviewer, NCBI (D1S3721, D1S2645, D1S2713, D1S3728) were used. These markers were amplified in a 20 μl reaction mixture containing 50 ng DNA, 0.2 μM of each primer, 0.2 mM dNTPs, 1×PCR buffer (Fermentas Inc., Maryland), 1.5 mM MgCl2 and 0.5 U Taq DNA polymerase (Fermentas Inc., Maryland). PCR condition was performed as follows: initial denaturation at 95°C for 5 minutes, followed by 35 cycles of denaturation at 95°C for 45 seconds, annealing at 60°C for 45 seconds, extension at 72°C for 45 minutes and a final extension at 72°C for 10 minutes. After PCR-amplification of genomic DNA samples, amplified fragments were resolved on an ABI PRISM 3100 Genetic Analyzer (Applied Biosystems, Foster City, CA) together with ROX-500 size standard (Applied Biosystems, Foster City, CA). The resulting genotype data were analyzed using GeneScan Analysis (Applied Biosystems, Foster City, CA). Allele numbers were assigned for each marker based on the size of the amplified fragment.

## Results

**Figure 2:**
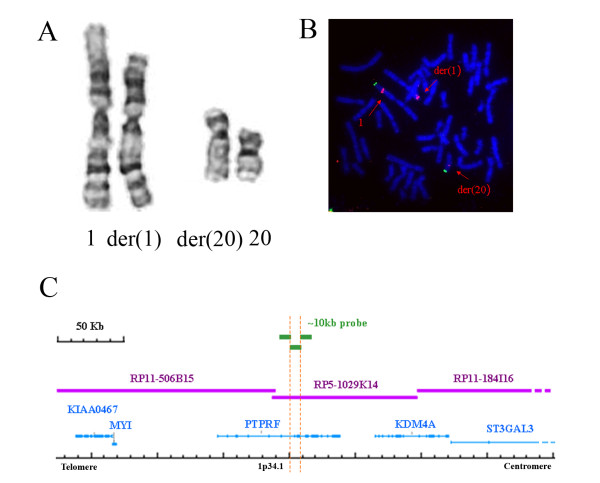
**Karyotype, FISH, and the breakpoint on chromosome 1**. (A) Partial karyotype shows apparently reciprocal balanced translocation of 1p34.1 and 20q13.13. (B) Fluorescence *in situ *hybridization using RP5-1029K14 labeled in red with spectrum orange dUTP, shows hybridization signals on both derivative chromosomes indicating that this probe encompasses the breakpoint. The 1p subtelomeric probe labeled with spectrum green dUTP indicates chromosome 1. (C) Schematic presentation of the clones and genes in the 1p34.1 region, modified from the NCBI Map Viewer http://www.ncbi.nlm.nih.gov/mapview. Three ~10 kb probes are shown in green numbered from left to right, BAC/PAC contigs covering the 1p34.1 breakpoint are shown in purple. The orange dotted lines indicate the critical region.

Karyotype analysis revealed an apparently reciprocal balanced translocation of t(1;20)(p34.1;q13.13) (Figure 2A). This similar translocation was also present in her unaffected mother and two unaffected family sisters except her elder brother showing normal karyotype of 46, XY.

Performing FISH, we identified the breakpoints on chromosomes 1 and 20 in the RP5-1029K14 and RP11-347D21 clones, respectively. While the RP11-347D21 clone did not contain any known genes, the RP5-1029K14 clone contained a gene, *Protein Tyrosine Phosphatase Receptor type F (PTPRF) *(MIM# 179590). We further narrowed the critical region, using the RP5-1029K14 clone as template and approximately 10-kb probes produced by long range PCR. One subfragment probe, covering intron 7 to intron 11 of the *PTPRF *gene, gave a split signal (Figure 2B, C). Analysis of the repetitive sequence elements in the vicinity of the breakpoint region found Alu, MIR (Mammalian Interspersed Repeat), LINE-L2, and ERVL-MaLRs (Endogenous Retrovirus; transposable element - Mammalian apparent LTR-Retrotransposon).

**Figure 3:**
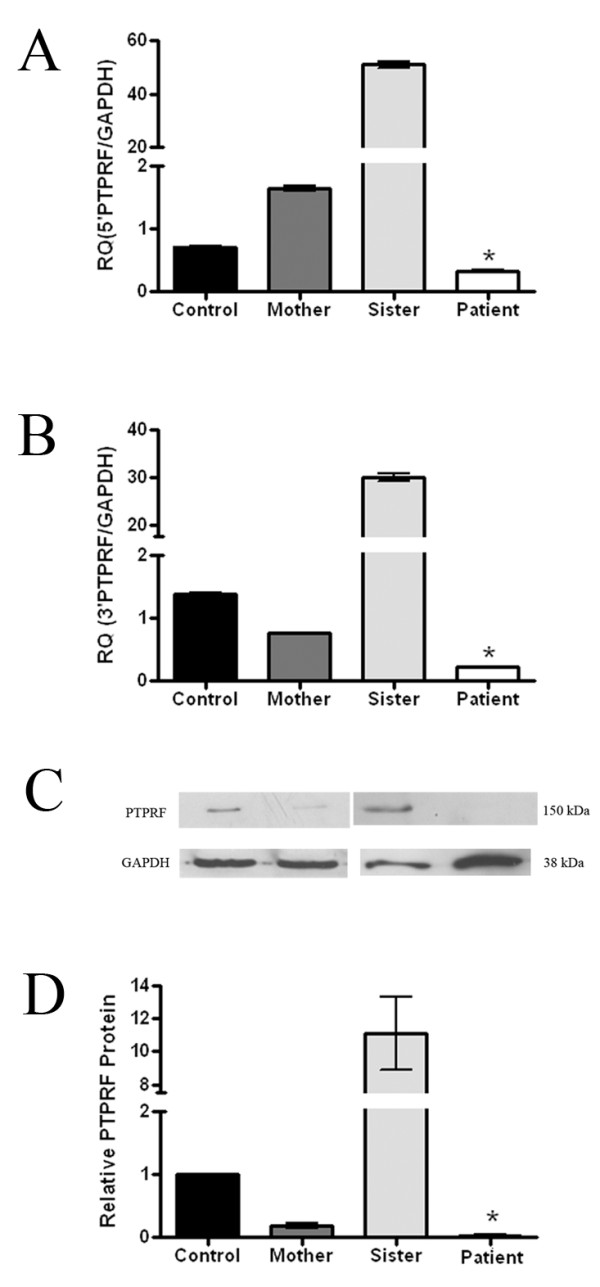
**Expression studies of the *PTPRF***. (A) Relative quantification using 5'*PTPRF *probe (proximal to the breakpoint) showed that the expression level in the proband was significantly decreased compared with that in a control, the proband's mother and sister. (B) Relative quantification using the 3'*PTPRF *probe (distal to the breakpoint) showed significantly decreased expression in the proband. Data are presented as mean ± SEM. ** indicates P < 0.01 (independent 1-tailed t-test) using *GAPDH *as a control. (C) Western blot analysis showing levels of the PTPRF protein (150 kDa) and GAPDH (38 kDa). (D) Western blot analysis indicates nearly absent PTPRF in the proband. Data are represented as mean ± SEM. * indicates P < 0.05 (independent 1-taliled t-test) using GAPDH as a control.

Quantitative real-time PCR analysis revealed that the *PTPRF*'s RNA level of the proband was significantly decreased (p < 0.01) compared with those of her mother, elder sister and a control (Figure 3A, B). RNA levels in the mother and elder sister were higher using the 5' probe compared with the 3' probe. In addition, the mother's *PTPRF *RNA level, determined by the 3' probe, was approximately half that of the control.

Western blot analysis revealed that PTPRF was nearly absent in the proband, while her unaffected mother and older sister had significant (p < 0.05) PTPRF protein remaining (Figure 3C, D).

Sequencing of the gene's entire coding regions and its promoter revealed many polymorphisms (Additional file 1, Table S5), but no obviously pathogenic mutations. In addition, reverse transcribed - PCR using RNA from peripheral leukocytes yielded no aberrant splicing patterns (data not shown).

Measurement of both DNA copy number and allelic ratios by High-density SNP genotyping array showed no evidence of deletions or duplications (Additional file 1, Figure S1). However, microsatellite analysis showed that the proband inherited a paternal chromosome different from that of her sisters (Additional file 1, Figure S2).

## Discussion

While studies in murine models revealed a crucial role of *ptprf *in mammary gland development, there has been no evidence of the *PTPRF *involvement in human breast development. Although, somatic mutations in the *PTPRF *gene have been found in many malignancies including breast cancer, its germline mutations have not been identified to cause any known human diseases.

We identified a woman with complete absence of both breasts and nipples along with ectodermal dysplasia and unilateral renal agenesis. Complete absence of breast is a very rare congenital anomaly. It can occur alone or with a wide range of associated features with a defect in ectoderm being the most common. It remains possible that some developmental stages of both ectodermal and breast tissues are influenced by the same gene.

Conventional cytogenetic analysis revealed an apparently reciprocal balanced translocation of t(1;20)(p34.1;q13.13) To our knowledge the t(1;20)(p34.1;q13.13) has not been observed in amastia patients or reported in any cases. Although this balanced translocation was also detected in her unaffected mother and sisters, there remained the possibility that the translocation breakpoint interrupted a gene responsible for breast development. Other groups previously identified *ALMS1 *as the gene responsible for the autosomal recessive disorder, Alström syndrome, based on evidence from a balanced chromosomal translocation. In that case, *ALMS1 *was interrupted by the translocation breakpoint, and the other allele had an intragenic mutation [7]. This stimulated us to determine whether any genes were interrupted by the breakpoints in our patient.

The FISH study revealed that the breakpoint on chromosome 1 was spanning from intron 7 to intron 11 of the *PTPR*F gene. Repetitive sequence elements, including Alu, MIR, LINE-L2, and ERVL-MaLRs were found in the vicinity of the breakpoint region. It is tempting to speculate that these transposable sequence elements have promoted this translocation. This situation is similar to other reported familial germline translocations which are also localized in regions rich in repetitive sequences [8].

*PTPRF*, also known as Leukocyte Common Antigen-Related molecule (*LAR*), is a member of the protein tyrosine phosphatase (PTP) family. This PTP contains an extracellular domain of three immunoglubulin-like domains in combination with eight fibronectin type-III-like repeats, a single transmembrane domain, and two intracellular tyrosine phosphatase domains [9]. *PTPRF *spans 92 kb, contains 34 exons, with an open reading frame of 5,724 bp, encoding a 1,907 amino-acid protein.

*Ptprf *knockout mice exhibit a defect in their mammary glands leading to the inability to deliver milk to their young [10]. In humans, *PTPRF *is recognized as a tumor suppressor gene; somatic mutations were identified in 9% of breast cancers [11], and some other malignancies showed significantly increased PTPRF expression [12]. However, germline mutations in *PTPRF *have not been identified and the gene has not been shown to influence organ development in humans.

To substantiate the role of *PTPRF *in human mammary gland development and to elucidate the mechanism underlying the phenotypic differences between the proband and her unaffected mother and sister with the same chromosome translocation, we studied the expression of the *PTPRF *in the family's EBV-transformed lymphoblastoid cell lines. RNA levels in the mother and elder sister were higher using the 5' probe (Figure 3A) compared with the 3' probe (Figure 3B), perhaps due to a feedback mechanism to increase transcription of both *PTPRF *alleles. If that were the case, then the presence of the breakpoint would give a lower level of intact *PTPRF *RNA when determined by the 3' probe [13]. As expected, the mother's *PTPRF *RNA level, determined by the 3' probe, was approximately half that of the control. However, the reason for the sister's elevated *PTPRF *RNA level remains to be elucidated.

We next determined PTPRF protein levels. Western blot analysis showed that PTPRF expression was nearly absent in the proband, while her unaffected mother and older sister had significant (p < 0.05) PTPRF protein remaining (Figure 3C, D), despite harboring the same balanced chromosomal translocation.

The proband's findings of severely deficient *PTPRF *RNA and absent PTPRF protein with an interrupted *PTPRF *allele on the maternal chromosome, suggested that our proband's paternal *PTPRF *allele harbored a pathogenic mutation. However, sequencing of the gene's entire coding regions and its promoter (-654 to + 294) revealed no obviously pathogenic mutations. Reverse transcribed - PCR yielded no aberrant splicing patterns, making a pathogenic intronic mutation or splice-site mutation unlikely. Measurement of both DNA copy number and allelic ratios showed no evidence of deletions or duplications (Additional file 1, Figure S1). However, microsatellite analysis showed that the proband inherited a paternal chromosome different from that of her sisters (Additional file 1, Figure S2). The reasons for significantly decreased *PTPRF *expression in our patient despite a lack of pathogenic mutations in the paternal allele remain to be elucidated.

## Conclusions

We have demonstrated that the *PTPRF *gene is interrupted on chromosome 1 in a woman with bilateral amastia and a balanced chromosome translocation. Although a definite pathogenic mutation on the paternal allele could not be identified, the patient's *PTPRF *RNA is severely deficient and PTPRF protein is barely detectable. While the proband's mother and sisters also harbor the translocated chromosome, their non-translocated chromosomes 1 are different from that of the proband and their PTPRF RNA and protein levels remain at significant levels. These findings make PTPRF a prime candidate to investigate for its spatial and temporal roles in human breast development.

## Consent

Written informed consent was obtained from the patient for publication of this case report and accompanying images. A copy of the written consent is available for review by the Editor-in-Chief of this journal.

## Competing interests

The authors declare that they have no competing interests.

## Authors' contributions

SA performed cytogenetic, molecular genetic and functional analyses and drafted the manuscript. ST performed molecular analyses and helped interpreting the results. CM, NR, and TS identified and diagnosed the patient. WG and TM helped and supported SNP array analysis. KS, VP and VS designed, supervised and participated in the editing of the manuscript. All authors read and approved the final manuscript.

## Pre-publication history

The pre-publication history for this paper can be accessed here:

http://www.biomedcentral.com/1471-2350/12/46/prepub

## Supplementary Material

Additional file 1 ***PTPRF *is disrupted in a patient with syndromic bilateral amastia**. Table S1. Review of the reported patients with bilateral amastia. Table S2. List of FISH probes and their results. Table S3. List of primers for generation of FISH probes using long-range PCR. Table S4. List of primers for mutation analysis of *PTPRF*. Table S5. List of *PTPRF *variations found in our patient with syndromic bilateral amastia. Figure S1. Result of SNP array of the patient. Figure S2. Haplotype analysis of chromosome 1 of family members Click here for file
