# Supplementary material for: PTPRF is disrupted in a patient with syndromic amastia
Source: BMC Med Genet. 2011 Mar 31;12:46. doi: 10.1186/1471-2350-12-46 (PMC3083333; doi:10.1186/1471-2350-12-46)
Supplement: Additional file 1 — PTPRF is disrupted in a patient with syndromic bilateral amastia. Table S1. Review of the reported patients with bilateral amastia. Table S2. List of FISH probes and their results. Table S3. List of primers for generation of FISH probes using long-range PCR. Table S4. List of primers for mutation analysis of PTPRF. Table S5. List of PTPRF variations found in our patient with syndromic bilateral amastia. Figure S1. Result of SNP array of the patient. Figure S2. Haplotype analysis of chromosome 1 of family members [file 1471-2350-12-46-S1.DOC]

**Additional file 1**

***PTPRF* is disrupted in a patient with syndromic bilateral amastia**

Surasawadee Ausavarat, Siraprapa Tongkobpetch, Verayuth Praphanphoj, Charan Mahatumarat, Nond Rojvachiranonda, Thiti Snabboon, Thomas C. Markello, William A. Gahl, Kanya Suphapeetiporn and Vorasuk Shotelersuk

**Table S1.** Review of the reported patients with bilateral amastia

| Number of cases | Prospect  Inheritance | Nipples | Ectodermal defect | Other anomalies | References |
| --- | --- | --- | --- | --- | --- |
| 23 | AD/AR | Hypoplastic in two cases; absent in others | Yes | deformities of hands and feet,  cleft palate,  absence of a finger,  saddle-nose,  hypertelorism | Trier, W.C., Plast Reconstr Surg, 1965. 36(4): p. 431-9. |
| 2 | AR | Absent | No | - | Kowlessar, M. and E. Orti, Am J Dis Child, 1968. 115(1): p. 91-2. |
| 1 | N/A | Absent | No | hearing defect,  malrotated kidneys | Tawil, H.M. and S.S. Najjar, J Pediatr, 1968. 73(5): p. 751-3. |
| 1 | N/A | Absent | No | spasticity, growth failure,  mental retardation,  strabismus,  bilateral simian creases | Mathews, J., N Y State J Med, 1974. 74(1): p. 87-9. |
| 1 | N/A | Absent | Yes | - | Burck, U. and K.R. Held, Clin Genet, 1981. 19(2): p. 117-21. |
| 3 | AD | Hypoplastic/ Absent | No | webbed fingers and toes,  polywhorls of scalp hair | Nelson, M.M. and C.K. Cooper, S Afr Med J, 1982. 61(12): p. 434-6. |
| 1 | AR | N/A | Yes | AREDYLD | Pinheiro, M., et al., Am J Med Genet, 1983. 16(1): p. 29-33. |
| 2 | AD | Hypoplastic/ Absent | YES | hypotrichosis, hyperpigmentation | Tsakalakos, N., et al., Arch Dermatol, 1986. 122(9): p. 1047-53. |
| 1 | N/A | Absent | No | ureteral triplication,  hypertelorism | Rich, M.A., et al., J Urol, 1987. 137(1): p. 102-5. |
| 1 | N/A | Absent | - | choanal atresia | Greenberg, F., Am J Med Genet, 1987. 28(4): p. 931-4. |
| 1 | N/A | N/A | Yes | AREDYLD | Breslau-Siderius, E.J., et al., Am J Med Genet, 1992. 44(3): p. 374-7. |
| 11 | AD | Hypoplastic/ Absent | Yes | scalp-ear-nipple syndrome,  hypertension | Edwards, M.J., et al., Am J Med Genet, 1994. 50(3): p. 247-50. |
| 1 | AD | Absent | Yes | scalp-ear-nipple syndrome,  renal hypoplasia  cataract  coloboma of the iris | Plessis, G., M. Le Treust, and M. Le Merrer, Clin Genet, 1997. 52(4): p. 231-4. |
| 1 | N/A | Absent | No | vaginal agenesis | Amesse, L., et al., Clin Genet, 1999. 55(6): p. 493-5. |
| 2 | N/A | Absent | Yes | scalp-ear-nipple syndrome,  renal and urinary tract anomalies | Picard, C., et al., Clin Genet, 1999. 56(2): p. 170-2. |
| 2 | AD | Absent | Yes | sensorineural hearing deficit,  dentinogenesis imperfecta, renal hypoplasia,  branchio-oto renal syndrome | Lin, K.Y., D.B. Nguyen, and R.M. Williams, Plast Reconstr Surg, 2000. 106(1): p. 98-101. |
| 1 | N/A | Absent | Yes | - | Iamin, M.T. and V.P. Kumar, Plast Reconstr Surg, 2003. 111(2): p. 959-61. |
| 1 | N/A | Hypoplastic | No | cardiovertebral alteration and absence of axillary hair | Martinez-Chequer, J.C., et al., Am J Obstet Gynecol, 2004. 191(1): p. 372-4. |
| 1 | N/A | Absent | Yes | - | Ligia, A.D., et al., Rev Chil Pediatr 2005. 76(2): p. 166-172. |
| 1 | N/A | Rudimentary | Yes | scalp-ear-nipple syndrome | Baris, H., W.H. Tan, and V.E. Kimonis, Am J Med Genet A, 2005. 134A(2): p. 220-2. |
| 1 | N/A | Absent | Yes | scalp-ear-nipple syndrome,  coloboma of the iris | Sobreira, N.L., et al., Am J Med Genet A, 2006. 140(3): p. 300-2. |
| 2 | AR | Hypoplastic | Yes | scalp-ear-nipple syndrome | Al-Gazali, L., et al., Clin Dysmorphol, 2007. 16(2): p. 105-7. |
| 1 | N/A | Absent | Yes | - | Klinger, M., et al., Case Report Med, 2009. 2009: p. 927354. |

N/A= not available

AD = Autosomal dominant

AR = Autosomal recessive

AREDYLD = Acral Renal Ectodermal Dysplasia Lipoatrophic Diabetes

**Table S2.** List of FISH probes and their results

| FISH probes | Position | Signal |
| --- | --- | --- |
| BAC/PAC clones |  |  |
| RP11-329N22 | 1p34.3 | 1p, der20 |
| RP5-1066H13 | 1p34.2 | 1p, der20 |
| RP11-282K6 | 1p34.1 | 1p, der20 |
| RP1-92O14 | 1p34.1 | 1p, der20 |
| RP11-506B15 | 1p34.1 | 1p, der20 |
| RP5-1029K14 | 1p34.1 | Split signal |
| RP11-184I16 | 1p34.1 | 1p, der1 |
| RP11-570P14 | 1p34.1 | 1p, der1 |
| RP11-30D7 | 1p34.1 | 1p, der1 |
| RP11-69J16 | 1p34.1 | 1p, der1 |
| RP11-291L19 | 1p34.1 | 1p, der1 |
| RP11-322N21 | 1p34.1 | 1p, der1 |
| RP11-49P4 | 1p33.0 | 1p, der1 |
| RP11-346M5 | 1p33.0 | 1p, der1 |
| RP11-330M19 | 1p33.0 | 1p, der1 |
| RP11-329A14 | 1p33.0 | 1p, der1 |
| RP11-428D12 | 1p33.0 | 1p, der1 |
| RP11-296A18 | 1p32.3 | 1p, der1 |
| RP11-334A14 | 1p32.3 | 1p, der1 |
| RP11-109I2 | 1p32.3 | 1p, der1 |
| RP11-243M12 | 1p32.2 | 1p, der1 |
| RP11-470E16 | 1p32.1 | 1p, der1 |
| RP11-32I17 | 1p31.3 | 1p, der1 |
| RP4-614O4 | 20q11.22 | 20q, der20 |
| RP11-425M5 | 20q11.23 | 20q, der20 |
| RP5-1123D4 | 20q12 | 20q, der20 |
| RP4-753D4 | 20q13.11 | 20q, der20 |
| RP1-47A22 | 20q13.11 | 20q, der20 |
| RP3-453C12 | 20q13.12 | 20q, der20 |
| RP5-1050K3 | 20q13.12 | 20q, der20 |
| RP4-569M23 | 20q13.13 | 20q, der20 |
| RP11-347D21 | 20q13.13 | Split signal |
| RP1-66N13 | 20q13.13 | 20q, der1 |
| RP5-1164I10 | 20q13.13 | 20q, der1 |
| RP4-791K14 | 20q13.13 | 20q, der1 |
| RP5-1114A1 | 20q13.2 | 20q, der1 |
| RP11-80K6 | 20q13.2 | 20q, der1 |
| RP4-749H19 | 20q13.31 | 20q, der1 |
| RP5-1018E9 | 20q13.32 | 20q, der1 |
| RP4-719C8 | 20q13.32 | 20q, der1 |
| RP11-157P1 | 20q13.33 | 20q, der1 |
| Probes generated from long-range PCR |  |  |
| RP5-1029K14-F1/R1 | 1p34.1 | 1p, der20 |
| RP5-1029K14-F2/R2 | 1p34.1 | Split signal |
| RP5-1029K14-F3/R3 | 1p34.1 | 1p, der1 |

**Table S3.** List of primers for generation of FISH probes using long-range PCR

| Primer Name | Primer sequence  (5’-3’) | Annealing temperature (°C) | Amplicon size (bp) |
| --- | --- | --- | --- |
| RP5-1029K14-F1 | GAG TGC GAT GAA GAT GAG GA | 59 | 8850 |
| RP5-1029K14-R1 | TCA GAC TGG TCT TGA ACT CC |
| RP5-1029K14-F2 | CCT GCT GCT GAC CTT GTG AC | 59 | 10050 |
| RP5-1029K14-R2 | CTG AGC ATC CAT CCA TAT GC |
| RP5-1029K14-F3 | CCT ATA GGA CCC AGT CAG GA | 57 | 8540 |
| RP5-1029K14-R3 | GTC ACA AGG TAA GCA GCA GG |

**Table S4.** List of primers for mutation analysis of *PTPRF*

| Primer Name | Primer sequence  (5’-3’) | Annealing temperature (°C) | Amplicon size (bp) |
| --- | --- | --- | --- |
| Promoter_PTPRF-F | TTG ATC TGG GAA TGG GAG AGC | 60 | 948 |
| Promoter_PTPRF-R | TGG ACT AGC GGG GAG GGC AAG G |
| PTPRF-EX3-F | CTG GAT GGT CAG TGA GGA TG | 57 | 560 |
| PTPRF-EX3-R | TCT GCA ACA GTG CAC ACA GC |
| PTPRF-EX4-5-F | TGA ACA GTG CCT GGC ACA TA | 57 | 750 |
| PTPRF-EX4-5-R | TCC ACC ACT GAC TTT CAC TG |
| PTPRF-EX6-F | TGC CTC TCA GAC CTG GAA AC | 61 | 700 |
| PTPRF-EX6-R | CAC CAC ACT GGC ACA TCA CA |
| PTPRF-EX7-F | CGT TGG TTC TAG ACA GGA GG | 57 | 630 |
| PTPRF-EX7-R | GCA CAT ACA CCA AGA AGT CG |
| PTPRF-EX8-F | GGC CTC AGT TTC CTA GGC TA | 63 | 792 |
| PTPRF-EX8-R | CTA GCA TGA TGT CTC CCA CC |
| PTPRF-EX9-F | CCT TCA GAG GTC ACC ATA AG | 63 | 922 |
| PTPRF-EX9-R | CCT AAC CTC ACA CCC TTA TC |
| PTPRF-EX10-11-F | TGT GTG GTC AGT TGG GAT GT | 58 | 1040 |
| PTPRF-EX10-11-R | AGT TCG GTT TGG CCA GCA GA |
| PTPRF-EX12-F | CAG CAC CTA AGG GGT AGC CT | 61 | 652 |
| PTPRF-EX12-R | CTT CTC CCA TCT TAG CCT GT |
| PTPRF-EX13-F | CAG GGA CAG ATG ATC TAA GG | 60 | 486 |
| PTPRF-EX13-R | AGC ATC TCG GGT CTC ACA AC |
| PTPRF-EX14-F | TCA GAG CAT CTG TAG CTG CT | 57 | 540 |
| PTPRF-EX14-R | CTG CTC GAC AGG CAA GAA GT |
| PTPRF-EX15-16-F | TCC TCT CCA GCA GAG GCC AC | 61 | 1054 |
| PTPRF-EX15-16-R | TTG GAG GTC ACA CAC CAG AG |
| PTPRF-EX17-19-F | CTT GGT ACT CTG CAG CCA TC | 57 | 947 |
| PTPRF-EX17-19-R | TGA GAG TCA CTG GGA CAG TG |
| PTPRF-EX20-21-F | GGG CTC TGA CAC GGA AGG TG | 61 | 958 |
| PTPRF-EX20-21-R | GGG TCC AGG ATG CAA GGC TG |
| PTPRF-EX22-F | GGG GCA GTA GGA GGA CAG AG | 59 | 301 |
| PTPRF-EX22-R | CCT GCA GAA CAG ACC CAC AG |
| PTPRF-EX23-F | GTG CTC CAT GGT CAC ACA TG | 59 | 280 |
| PTPRF-EX23-R | CTA GGA CAG GAC AGG AGC TG |
| PTPRF-EX24-25-F | TGG CTG GCA CCA CGA GAT AG | 57 | 720 |
| PTPRF-EX24-25-R | AGC GAC TTC CTC CAC AGA AG |
| PTPRF-EX26-27-F | GGT GAC ATA GCT TGA GGA CC | 60 | 840 |
| PTPRF-EX26-27-R | GCA GGA CAC GGG CTC AGC TT |
| PTPRF-EX28-29-F | ACT GCA GGT GAG AGG GTA CA | 59 | 770 |
| PTPRF-EX28-29-R | CCC TCT CTG GTC TTC TAG GC |
| PTPRF-EX30-31-F | TAG AGC AGT GAG GAC TTC CT | 57 | 693 |
| PTPRF-EX30-31-R | CTT GAG CTA AGG TAC CAT GC |
| PTPRF-EX32-33-F | CAT GGT ACT ACC CTG GTC TA | 57 | 640 |
| PTPRF-EX32-33-R | GGT AGG ACC ATA AGC ACA CA |
| PTPRF-EX34-F | CTA ACT CCA TGG CTG CAG TG | 57 | 269 |
| PTPRF-EX34-R | CCA GTG ACA GCA TCT GCG TA |
| PTPRF-mRNA-F1 | TGG ATA GGC GGA AGG AGT GG | 60 | 993 |
| PTPRF-mRNA-R1 | CTC CAG GAC GTT GCG GCC AA |
| PTPRF-mRNA-F2 | TGC TCG AAG AGG AAC AGC TG | 60 | 760 |
| PTPRF-mRNA-R2 | CCA ATG CTG TAG CGG GTG GT |
| PTPRF-mRNA-F2.1 | CAG CGT GAA CCT GAC ATG CG | 60 | 1298 |
| PTPRF-mRNA-R2.1 | ACT CCG TCC ACT TCT CCA GG |
| PTPRF-mRNA-F3 | CAG AAG GTG ATG TGT GTG AG | 60 | 1050 |
| PTPRF-mRNA-R3 | GCT GTT GAT GTC TCG GAA C |
| PTPRF-mRNA-F4 | TCG AGA AGG AGA TCA GGA CC | 60 | 1280 |
| PTPRF-mRNA-R4 | TGG GTG GTC TCG CAT ACC TG |
| PTPRF-mRNA-F5 | CCC TAC TCG GAT GAG ATC GT | 52 | 1330 |
| PTPRF-mRNA-R5 | GCA GAC ACA CAC GGG TCA AT |
| PTPRF-mRNA-F6 | GGA GCG GAT GAA GCA CGA GA | 60 | 1456 |
| PTPRF-mRNA-R6 | CAA CCC CTA CAG TGG CCC AT |
| PTPRF-mRNA-F7 | GTA CCT CGG CAG CTT TGA CC | 60 | 1570 |
| PTPRF-mRNA-R7 | CAG CAC TAG CAT CCA CAA GG |

**Table S5.** List of *PTPRF* variations found in our patient with syndromic bilateral amastia

| Position | Reference SNP | Genotype |
| --- | --- | --- |
| Promoter | Unreported | +92delGGCTCC |
| Intron 5 | rs2842185 | TT |
| Intron 5 | rs2842186 | AG |
| Exon 6 | rs1065771 | CC |
| Intron 6 | rs943513 | GG |
| Intron 6 | rs11210874 | TC |
| Intron 9 | rs2304353 | GG |
| Intron 9 | g.60814 G>T | GG |
| Intron 10 | rs7540068 | GT |
| Intron 10 | rs7553297 | AG |
| Exon 11 | rs3828151 | CA |
| Intron 14 | rs12023161 | AG |
| Intron 17 | rs17371903 | AA |
| Exon 19 | rs631248 | GA |
| Exon 20 | rs1065772 | CT |
| Exon 20 | rs10890266 | CT |
| Intron 20 | rs539096 | AG |
| Intron 23 | rs603542 | CT |
| Intron 23 | rs571862 | CT |
| Exon 25 | rs641365 | TC |
| Exon 25 | rs641351 | GA |
| Exon 27 | rs1143701 | CT |
| Intron 30 | rs568639 | TC |
| Exon 33 | rs1143702 | CT |

**Figure S1.** Result of SNP array of the patient


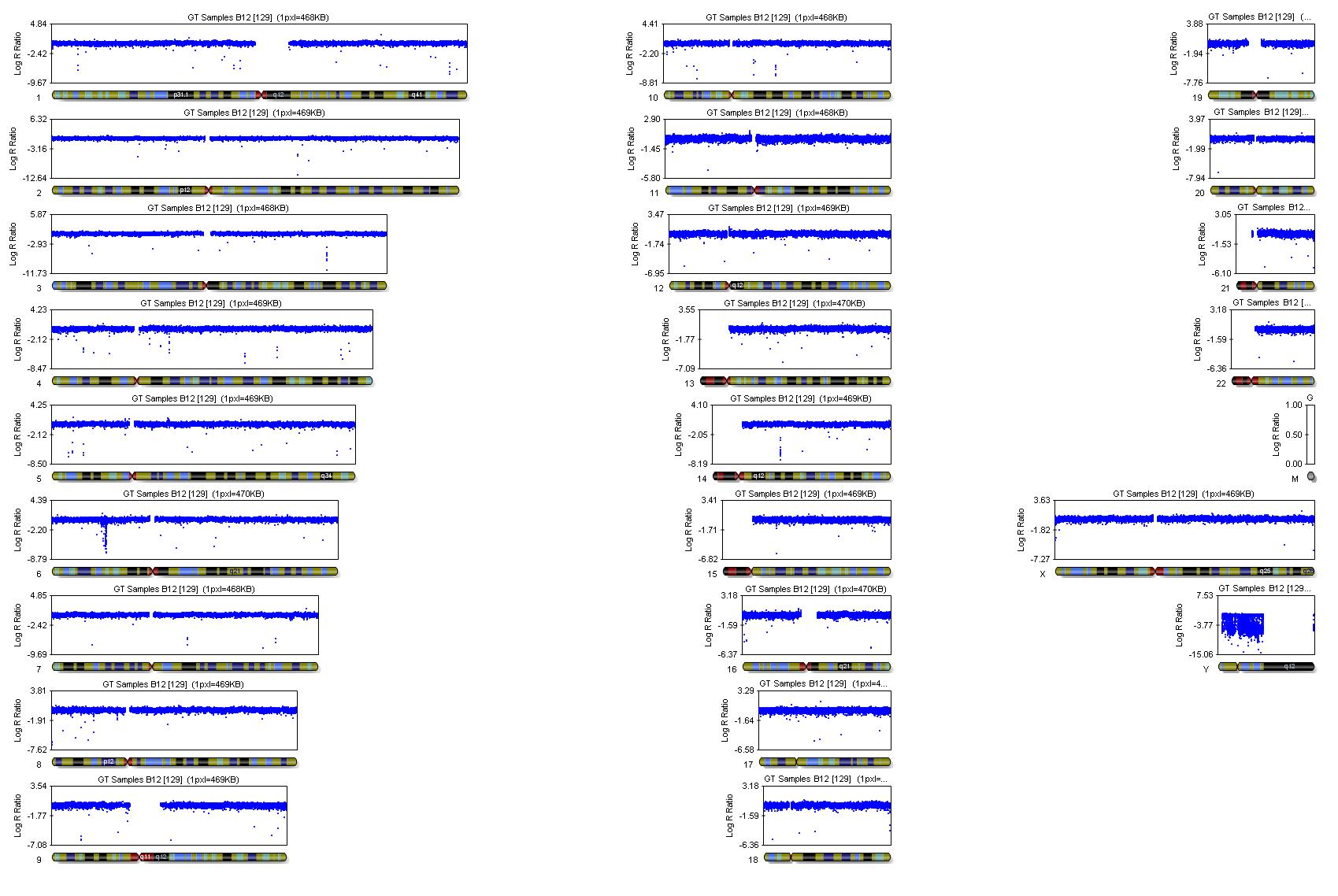


**Figure S2.** Haplotype analysis of chromosome 1 of family members.


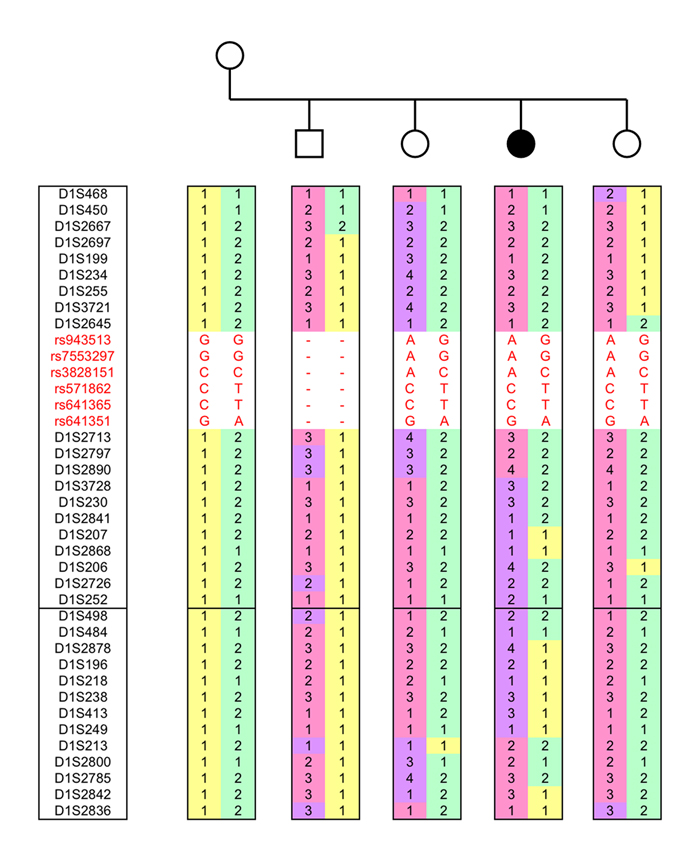


Two vertical boxes on each row indicate the border of 1p and 1q, respectively. Red color markers indicate the intragenic SNP. Haplotype of each member was imputed. Colors of each row represent the predicted inherited haplotype.
